# Protein subnuclear localization based on a new effective representation and intelligent kernel linear discriminant analysis by dichotomous greedy genetic algorithm

**DOI:** 10.1371/journal.pone.0195636

**Published:** 2018-04-12

**Authors:** Shunfang Wang, Yaoting Yue

**Affiliations:** School of Information Science and Engineering, Yunnan University, Kunming, PR China; Northeast Normal University, CHINA

## Abstract

A wide variety of methods have been proposed in protein subnuclear localization to improve the prediction accuracy. However, one important trend of these means is to treat fusion representation by fusing multiple feature representations, of which, the fusion process takes a lot of time. In view of this, this paper novelly proposed a method by combining a new single feature representation and a new algorithm to obtain good recognition rate. Specifically, based on the position-specific scoring matrix (PSSM), we proposed a new expression, correlation position-specific scoring matrix (CoPSSM) as the protein feature representation. Based on the classic nonlinear dimension reduction algorithm, kernel linear discriminant analysis (KLDA), we added a new discriminant criterion and proposed a dichotomous greedy genetic algorithm (DGGA) to intelligently select its kernel bandwidth parameter. Two public datasets with Jackknife test and KNN classifier were used for the numerical experiments. The results showed that the overall success rate (OSR) with single representation CoPSSM is larger than that with many relevant representations. The OSR of the proposed method can reach as high as 87.444% and 90.3361% for these two datasets, respectively, outperforming many current methods. To show the generalization of the proposed algorithm, two extra standard datasets of protein subcellular were chosen to conduct the expending experiment, and the prediction accuracy by Jackknife test and Independent test is still considerable.

## Introduction

Subnuclear localization of protein is very important for molecular cell biology, proteomics, and drug discovery and so on [1, 2]. When the basic function of a protein is known, the information about its location in the cell nucleus may indicate some important facts such as the pathway an enzyme belongs to [2]. Thus, if proteins are located at wrong positions in the nucleus or in a cell, some diseases, even cancer, will be caused. With the development of human genome project and proteomics project, numerous protein sequences increase dramatically day by day so that those traditional experimental methods can’t satisfy the demands of current researches on account of their low efficiency and highly cost. Therefore, in order to manage and address these huge biological data, computational techniques are essential. There are two typical procedures when researchers apply machine learning methods to predict protein subnuclear location. One is to construct good representations for collecting as much protein sequence information as possible and the other is to develop effective models for prediction and classification [3, 4].

As far as feature representations are concerned, Nakashima and Nishikawa proposed a well-known representation, amino acid composition (AAC) [5], which describes the occurrence frequency of 20 kinds of essential amino acids in a protein sequence. However, AAC ignores the associated information among amino acids [4]. Therefore, dipeptide composition (DipC) was presented by considering 400 components of dipeptide composition information along local order of amino acids [6]. Nevertheless, the discrimination of DipC is still insufficient. Subsequently, taking into account both amino acid composition information and amphipathic sequence-order information, Chou et al. introduced the pseudo-amino acid composition (PseAAC), and relevant experimental results proved that the discriminant performance of PseAAC overmatched both AAC and DipC partly [7–11]. Afterwards, the position-specific scoring matrix (PSSM) was proposed by considering the evolution information of amino acids, and PSSM is more helpful than PseAAC for protein subnuclear localization [12]. But the prediction accuracy still can't meet researchers’ expectation. Hence, they tried to build more efficient protein feature expression. Based on above single feature representations, researchers proposed the concept of fusion representation by combining two single expressions for improving the prediction accuracy since fusion representation contained more original protein sequence information [4, 13, 14]. However, although the predictive accuracy is improved with this kind of method, the extra workload and time-consuming caused by the process of fusing different representations increase a lot [4]. With this consideration, the research of this paper devoted to developing a new single representation to make it can express protein sequence more effectively, and then lots of time will be saved by doing so relative to the fusion representation.

Next, due to the high dimensionality of protein feature representation data, lots of dimension reduction algorithms were employed to extract feature such as linear discriminate analysis (LDA) [4, 13, 15], principal component analysis (PCA), kernel principal component analysis (KPCA) [12], kernel entropy component analysis (KECA), kernel LDA (KLDA) and so on [12, 16–22]. Since the nonlinear characteristics are more popular than the linear characteristics in biology [12, 23, 24], the nonlinear kernel algorithms are the keystone of this paper. Especially, KLDA is selected to use because it not only can reduce dimensionality but also can help classification and recognition. However, the window width parameter of kernel function in current studies tends to be empirically selected, which is not reasonable. So instead, in this paper, we realized the intelligent selection of the bandwidth parameter by our proposed new discriminant criterion and optimization algorithm DGGA. After extracting good features, researchers try to develop effective classifiers for prediction, including biological neural networks, Bayesian networks, support vector machines (SVM), ensemble-classifiers [25, 26], optimally weighted fuzzy K-NN algorithm [2] and so on. Thereinto, the neural network not only needs mass of data to train the structural classifier model, but also needs effective method and plenty of time to tune the network parameters, which is a hard problem to be improved [27]. Similarly, the prediction results based on Bayes discriminant algorithm can be improved as the amount of training data increases; therefore, a small number of training data may cause the prediction results less stable [28]. In addition, although the SVM, ensemble-classifier and the weighted fuzzy K-NN algorithm can get good prediction, they are all time-consuming in the training phase [25, 29–31]. Compared with them, the KNN classifier is lazy learning, namely, it almost doesn't have to train, which will save a certain amount of time. In practical application, the calculated quantity of KNN classifier is proportional to the sample size [32]. Here in this paper, the experimental data are small sample size, which makes it more appropriate to utilize the KNN classifier compared to those classifiers mentioned above. Hence, we only employ the simple KNN classifier in this paper to both reduce the computational complexity and highlight the innovation of the proposed method, CoPSSM with intelligent KLDA based on DGGA.

To sum up, although good results were obtained based on those above approaches, namely, fusing different representations, developing more effective models or classifiers, shortcomings still exist in current works. Computation complexity, for instance, increases a lot to some extent. So, if we can improve the prediction accuracy of protein subnuclear location only use the single feature representation and simple classifier, a lot of time-consuming and costly work will be saved, and that will be very meaningful. And here, the work of this paper is just to realize this goal. First of all steps, the single feature vector, position-specific scoring matrix (PSSM), was extracted from the given original protein sequence and then a new feature expression CoPSSM would be created based on the PSSM matrix. Next, the nonlinear dimensionality reduction (DR) algorithm, kernel linear discriminant analysis (KLDA), whose bandwidth parameter was intelligently optimized by the proposed new discriminant criterion and dichotomous greedy genetic algorithm (DGGA), has been employed to address the high-dimensionality problem by transforming the representation of protein sequence for arriving at an optimal expression for K-nearest-neighbor (KNN) classifier. The final numerical experimental results with Jackknife test show our proposed single feature representation CoPSSM and optimization algorithm are efficient in the prediction of protein subnuclear location.

Here, we listed abbreviation of the full name for all terms appeared in this paper in Table 1.

**Table 1:** Abbreviation for the corresponding term.

Number	Full Name of Term	Abbreviation
1	Pseudo-amino acid composition	PseAAC
2	Position-specific scoring matrix	PSSM
3	Correlation position-specific scoring matrix	CoPSSM
4	Kernel linear discriminant analysis	KLDA
5	K-nearest-neighbor	KNN
6	Genetic algorithm	GA
7	Dichotomous greedy genetic algorithm	DGGA
8	Overall success rate	OSR
9	True positive	TP
10	True negative	TN
11	False positive	FP
12	False negative	FN
13	Sensitivity	SE
14	Specificity	SP
15	Accuracy	ACC
16	Mathew’s correlation coefficient	MCC

## Materials and methods

### Dataset

To validate the adaptability and the efficiency of the proposed method in this paper, and to have a critical comparison with other studies, two public benchmark datasets were chosen to conduct the numerical experiments. The first dataset was constructed by Ravindra Kumar et al. in 2014 [33], which is in their web-server named SubNucPred for predicting protein sub-nuclear localization with the link of http://proteininformatics.org/mkumar/subnucpred/index.html. The second dataset is in web-sever Nuc-Ploc [7], which was constructed by Shen and Chou in 2007 and could be downloaded from the link of http://www.csbio.sjtu.edu.cn/bioinf/Nuc-PLoc/Supp-A.pdf. Detailed information of these two datasets is in Table 2.

**Table 2:** Constitutions of protein benchmark datasets.

Dataset 1 of ten subnuclear locations			Dataset 2 of nine subnuclear locations		
Class	Subnuclear Location	Number	Class	Subnuclear Location	Number
1	Centromere	86	1	Chromatin	99
2	Chromosome	113	2	Heterochromatin	22
3	Nuclear envelope	17	3	Nuclear envelope	61
4	Nuclear matrix	18	4	Nuclear matrix	29
5	Nuclear pore complex	12	5	Nuclear pore complex	79
6	Nuclear speckle	50	6	Nuclear speckle	67
7	Nucleolus	294	7	Nucleolus	307
8	Nucleoplasm	30	8	Nucleoplasm	37
9	Telomere	37	9	Nuclear PML body	13
10	Nuclear PML body	12			
**Sum**		**669**	**Sum**		**714**

As shown in Table 2, dataset 1 totally contains 669 proteins that attribute to 10 subnuclear localizations and dataset 2 contains 714 proteins in total and locates at 9 subnuclear localizations.

Next, to show the generalization of the proposed method, two protein subcellular benchmark datasets were chosen to conduct the expending experiment, as are shown in Table 3.

**Table 3:** Constitutions of protein benchmark datasets for expending experiment.

Class	Subcellular Location	Number of Dataset 3	Number of Dataset 4
1	Cytoplasm	152	210
2	Extracell	76	20
3	Fimbrium	12	4
4	Flagellum	6	1
5	Inner membrane	186	345
6	Nucleoid	6	1
7	Outer membrane	103	13
8	Periplasm	112	49
**Sum**		**653**	**643**

From Table 3, it can be found that datasets 3 and 4 contain 653 and 643 proteins, respectively, and they can be downloaded directly from http://www.csbio.sjtu.edu.cn/bioinf/Gneg/Data.htm [34].

To sum up, in this paper, we used four datatsets that were constructed in previous studies [7, 33, 34]. For an easy access to all these data, we construct a new link to gather all the link information about these datasets, that is https://github.com/tingyaoyue/Dataset.git.

### A newly proposed feature representation CoPSSM

Before introducing the proposed new feature expression CoPSSM, we first need to give some brief presentation for PseAAC and PSSM that are used for comparison in this paper. Then, introduction for CoPSSM will be deployed based on PSSM.

1. PseAAC, put forward by Chou et al., represents a protein sequence with its sequence composition and order information in a vector [7]. Generally speaking, PseAAC is expressed as *P*_*PseAAC*_ = [*p*_1_,*p*_2_,…,*p*_20_,*p*_20+1_,…,*p*_20+2*β*_]^*T*^. And here, the parameter *β* is set as 10 empirically to obtain a 40-D feature vector. The first 20 components reflect the effect of the classical 20 amino acid composition, and components from 20 + 1 to 20 + 2*β* reflect the amphipathic sequence-order pattern with considering the impact of hydrophobic and hydrophilic of amino acids [35–38].
2. PSSM, whose description is as below:
  A variety of variations, such as the insertion, substitution or deletion of one or several amino acid residues in the protein sequence, often occur in the biological evolution process. And with long-term accumulation of these variations, similarities between the original and the new synthesis proteins are reducing gradually, but these homologous proteins may exhibit remarkably similar structures and functions [39]. Hence, the position-specific scoring matrix (PSSM) was introduced to represent the evolution information of a protein sample P with L amino acid residues. Its descriptor is shown as following:
  $$P_{PSSM}=\left[\begin{array}{cccc} M_{1\to 1} & M_{1\to 2} & \cdots & M_{1\to 20}\\ M_{2\to 1} & M_{2\to 2} & \cdots & M_{2\to 20}\\ \vdots & \vdots & \ddots & \vdots \\ M_{L\to 1} & M_{L\to 2} & \cdots & M_{L\to 20} \end{array}\right]$$ (1)
  where *M*_*i*→*j*_(*i* = 1,2,…,*L*; *j* = 1,2,…,20) represents the score of the amino acid residue in the *i th* position of the protein sequence being replaced by the amino acid type *j* during the evolution process. And here in this paper, the *P*_*PSSM*_ matrix was generated via using PSI-BLAST to search the Swiss-Prot database, of which the iterative times were 3 and the E-value was 0.001.
3. Introduction for the newly proposed feature representation CoPSSM

Feature representation plays an important role in protein subnuclear localization [4]. Based on this idea, this paper skillfully proposed a new feature expression. Since sizes of above obtained *P*_*PSSM*_ matrices were not unified for different proteins, researchers usually transformed them into 400-D vectors by adding all rows of the same element [12, 13]. Here, we will develop a better representation.

Firstly, calculate average value of each column in *P*_*PSSM*_ according to (2).

$${\bar{M}}_{j}=\frac{1}{L}\sum_{i=1}^{L}M_{i\to j}\;\left(j=1,2,\ldots 20\right)$$ (2)

Secondly, calculate the product of two different elements in the above obtained 20 average values according to formula (3).

$$CoPSSM={\bar{M}}_{j}∙{\bar{M}}_{k}\;\left(j=1,2,\ldots ,20;\;k\;is\;integer\;and\;j\leq k\leq 20\right)$$ (3)

Last, a 210-D vector shown in (4) will be attained according to Eqs (2) and (3). Since it takes the correlation of two different average values into consideration, we named the proposed feature representation as correlation position-specific scoring matrix CoPSSM.

$$CoPSSM=\left(CoPSSM_{1},\;CoPSSM_{2},\ldots ,\;CoPSSM_{210}\right)$$ (4)

### A newly proposed discriminant criterion added to KLDA

Kernel linear discriminant analysis, also known as generalized discriminant analysis is the kernel extension method of linear discriminant analysis (LDA), which is expanded to solve the nonlinear problems. Therefore, KLDA is much suitable for processing biological data because of its high-dimensional and nonlinear characteristics. KLDA algorithm maps the input vectors to a higher dimensional feature space *F* via the nonlinear mapping function ∅, and then it executes the linear discriminant analysis in the high dimensional feature space [40]. To increase further understanding, the KLDA algorithm will be described in detail in the S1 File, in which, these two literatures [41, 42] will be cited.

But what actually matters is the bandwidth parameter of the kernel function, which changes the mapping relation between the input space and the feature space so that it can affect the properties of the feature space. So far, there is a lack of good methods to find out the best value of the window width parameter [40, 43]. Usually, researchers set this parameter empirically. Besides, a method called grid searching method was used to determine value of this parameter [44]. But they were partly irrational for lacking of a rational answer even good results were obtained, and there still existed defects in the grid searching method, missing the valid values and much time-consuming for instance. Thus, we try to introduce more reasonable method to select the bandwidth parameter to deal with this problem in protein subnuclear location. Here in this paper, the gauss kernel function: $K(x,y)=exp(-\frac{{\left\|x-y\right\|}^{2}}{2\sigma^{2}})$ is taken into consideration, and we’ll propose a new discriminant criterion to evaluate its bandwidth parameter σ whether good or not.

Providing that the reduced samples by KLDA are *r*_1_,*r*_2_,…,*r*_*N*_, then we define a new discriminant criterion to evaluate distinguishability of these reduced data, as formula (5):

$$\max\;\frac{D_{B}}{D_{W}}$$ (5)

where *D*_*W*_ and *D*_*B*_ represents the within-class dispersion and the between-class dispersion, respectively. Our purpose here is to minimize *D*_*W*_ and to maximize *D*_*B*_. Nevertheless, they two will change randomly during the course of the experiment. Hence, we defined the above ratio and stipulated that the best reduced data corresponded to the largest ratio. Hence, formula (5) is set as the fitness function of the proposed dichotomous greedy genetic algorithm (DGGA). Thereinto, *D*_*W*_ and *D*_*B*_ can be obtained via formulas (6) and (7).

$$D_{W}=\sum_{i=1}^{K}\sum_{m=1}^{N_{i}}\|r_{m}^{i}-a_{i}\|{}_{2}$$ (6)

$$D_{B}=\sum_{i=1}^{K}\|a_{i}-a{\|}_{2}$$ (7)

where the 2-norm ‖∙‖_2_ denotes the Euclidean distance, *K* is the number of protein type, *N*_*i*_ is the number of class *i*, *a*_*i*_ represents the mean vector of class *i* and *a* denotes the mean vector of all classes, i.e., $a_{i}=\frac{1}{N_{i}}\sum_{m=1}^{N_{i}}r_{m}^{i}$ and $a=\frac{1}{N}\sum_{n=1}^{N}r_{n}$.

### An improved dichotomous greedy genetic algorithm (DGGA) for kernel parameter selecting

Genetic algorithm (GA) is a kind of adaptive method for dealing with complex optimization problem whose core is the “survival of the fittest” rule and chromosomal crossover mechanism within a group. Increasingly wide attention and applications are drawn in GA for its robustness, parallelism and global optimization characteristics in recent years. Whereas shortcomings still exist in this algorithm such as easy to trap in local optimum. Therefore, we proposed a new algorithm, dichotomous greedy genetic algorithm (DGGA), to improve general GA algorithm. DGGA is based on GA by introducing the idea of inter-partition and Greedy Algorithm. In simple terms, in order to search the gauss kernel parameter more efficiently, we keep on dividing the interval into two subintervals and reserving the effective one on which the largest fitness is obtained by employing GA, which derives from the theory of Greedy Algorithm until the iterations run out. Thus, the proposed DGGA is named as dichotomous greedy genetic algorithm, of which, the word dichotomous means dividing the interval into two subintervals continually and greedy signifys the using of the idea of Greedy Algorithm. The specific steps of DGGA are listed as below.

- Step1: Select a certain amount of points randomly in the given interval [*X*_0_,*X*_*n*_], as the initial population;
- Step2: Calculate fitness of the initial population;
- Step3: Let *X*_*max*_ be the location identifier of the point with maximal fitness among the initial population, then we get 2 inter-partitions [*X*_0_,*X*_*max*_] and [*X*_*max*_,*X*_*n*_];
- Step4: Generate the initial population *P*_1_ and *P*_2_ randomly in the inter-partition of [*X*_0_,*X*_*max*_] and [*X*_*max*_,*X*_*n*_] respectively, then calculate their fitness respectively, named *f*_1_ and *f*_2_;
- Step5: Employed GA to optimize the kernel parameter with (*f*_1_,*P*_1_) and (*f*_2_,*P*_2_) as the input parameters respectively, then the updated population and fitness are marked as (*newf*_1_, *newP*_1_) and (*newf*_2_, *newP*_2_);
- Step6: Let *maxfit*_1_ be the max value of *newf*_1_ and *maxfit*_2_ be the max value of *newf*_2_.
- Step7: If *maxfit*_1_ is larger than *maxfit*_2_, let *X*_1_ be the corresponding location identifier and make *X*_*n*_ = *X*_*max*_, then let *X*_*max*_ = *X*_1_; else, let *X*_2_ be the corresponding location identifier of *maxfit*_2_ and make *X*_0_ = *X*_*max*_, then let *X*_*max*_ = *X*_2_. Therefore, the updated inter-partitions are as [*X*_0_,*X*_*max*_] and [*X*_*max*_,*X*_*n*_]. Next, turn to Step4 until the iterations run out.

**Fig 1:**
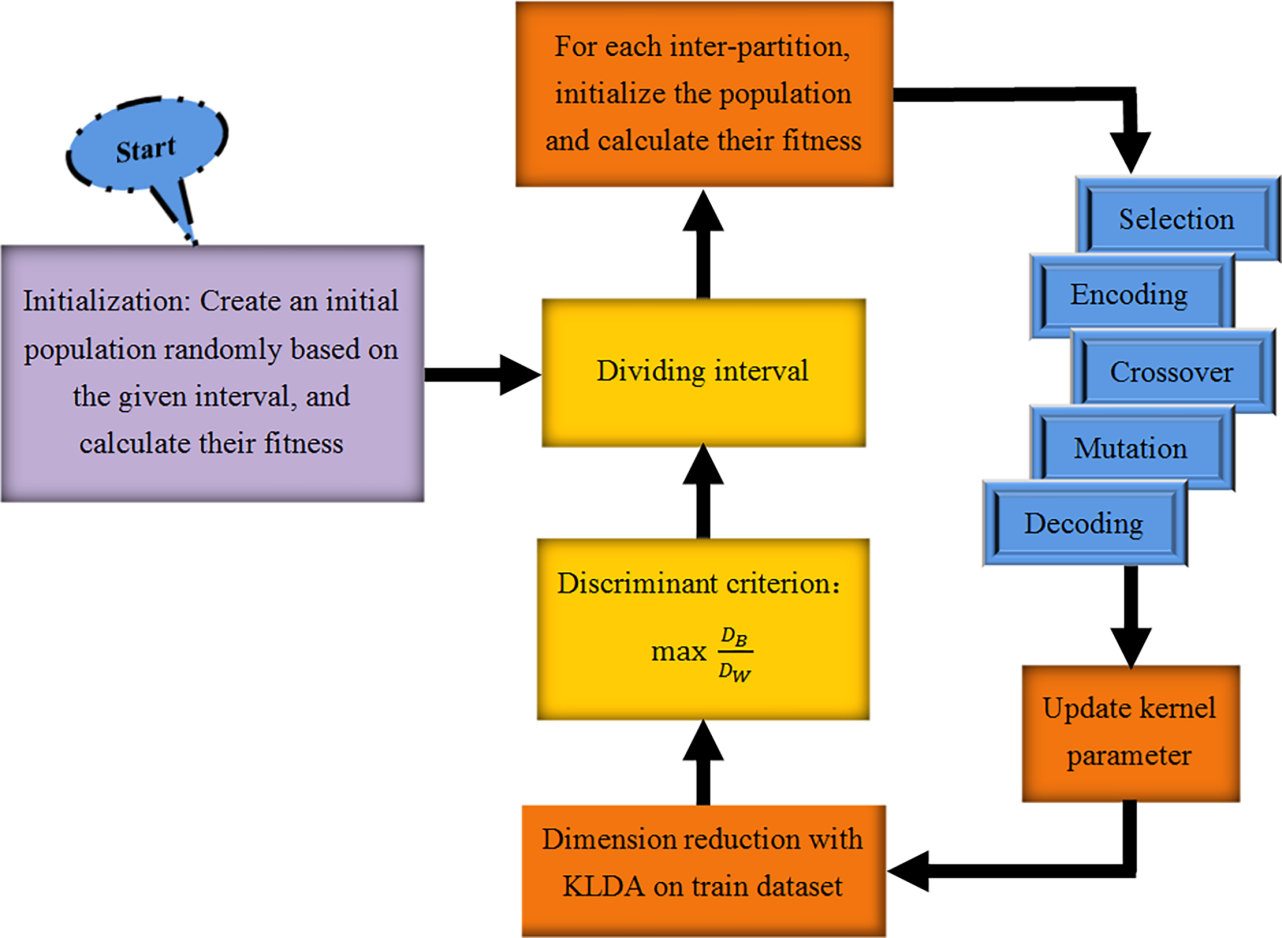
The DGGA algorithm for searching kernel parameter.

At last, to have an intuitivism apprehension of DGGA, we summarize the above procedures and give the full design flow of DGGA for optimizing the kernel parameter, displayed in Fig 1.

**Fig 2:**
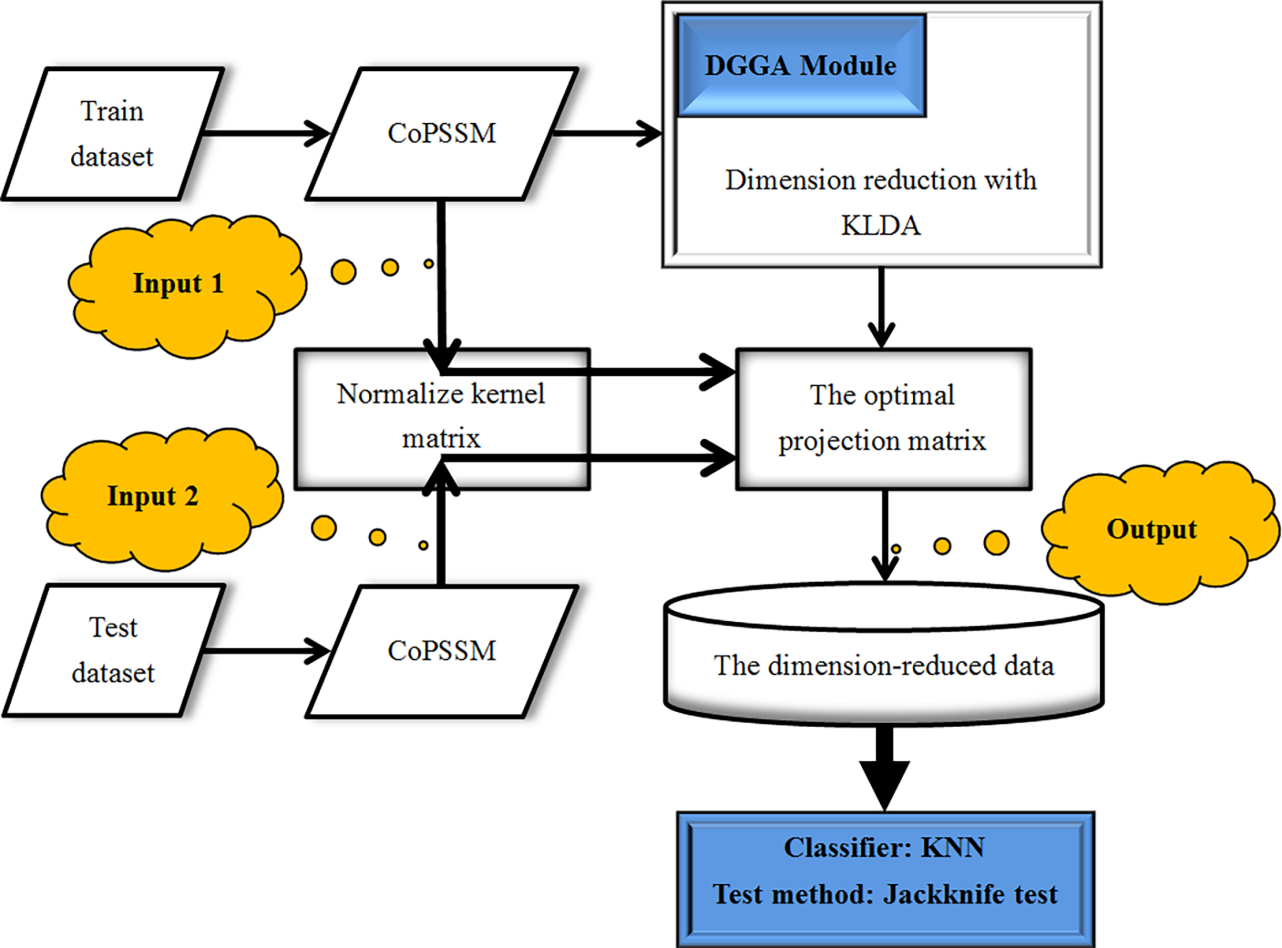
The flowchart for predicting protein subnuclear location.

After the optimal kernel parameter had been trained, it would be realizable to calculate the optimal projection matrix, and then both train dataset and test dataset would be mapped to the low-dimensional feature space. Last, the KNN classifier would be employed to predict corresponding protein subnuclear location according to the rule of Jackknife test. We’ll provide Fig 2 to display specific flows of the whole processes predicting protein subnuclear location.

### Assessment criteria, classifier and test method

To evaluate prediction performance of the proposed method, indexes: Sensitivity (SE), Specificity (SP), ACC (Accuracy) and MCC (Mathew’s Correlation Coefficient) are calculated to compare different representations in the case of Jackknife test. In the following formulas (8)–(12), TP means the true positive and TN means the true negative, of which both are the number of proteins that were correctly located, while FP (the false positive) and FN (the false negative) are the number of those that were wrongly located proteins[4]. Then, 4 index equations are obtained:

SE(i)=TP(i)/(TP(i)+FN(i))(i=1,2,…,k) (8)

SP(i)=TN(i)/(TN(i)+FP(i))(i=1,2,…,k) (9)

$$ACC(i)=\frac{TP(i)+TN(i)}{TP(i)+FP(i)+TN(i)+FN(i)}\;(i=1,2,\ldots ,k)$$ (10)

$$MCC(i)=\frac{\left(TP(i)\cdot TN(i))-(FP(i)\cdot FN(i)\right)}{\sqrt{\left(TP(i)+FP(i))\cdot (TP(i)+FN(i))\cdot (TN(i)+FP(i))\cdot (TN(i)+FN(i)\right)}}\;(i=1,2,\ldots ,k)$$ (11)

Here, SE, also called the success rate, denotes the rate of positive samples correctly located; SP denotes the rate of negative samples correctly located and ACC means the rate of correctly located samples. MCC returns a value lying in [–1, 1] and the value of MCC reflects the prediction consequences. The value of 1 denotes a perfect prediction, 0 represents random prediction and -1 represents a bad prediction. Generally, MCC is regarded as one of the best assessment indexes [45].

In addition, we also defined the overall success rate (OSR) as follow to evaluate the overall classification effects. From Eqs (8) to (12), *k* is the number of protein type.

$$OSR=\frac{\sum_{i=1}^{k}TP(i)}{\sum_{i=1}^{k}\left(TP(i)+FN(i)\right)}$$ (12)

Last, in this paper, we take KNN as the classifier for its simplicity, but competitive results. The Cosine distance is used to measure the close degree of two proteins. Besides, Jackknife test which is accounted as the most reasonable testing method are employed to estimate the prediction performance of our proposed method.

## Results and discussion

In this paper, the number of the initial population and the iterations were selected as 10 with taking the calculating time into consideration. Larger population and bigger iterations will cost more time undoubtedly, while smaller population and iterations may cause incomplete optimization. Next, we empirically set probability of the selection, crossover and mutation operator as 0.5, 0.7 and 0.1 respectively. Finally, the hardware operating environment of this paper is: Intel(R) Core(TM) i7-3770 CPU @3.40GHz 3.40 GHz, RAM 4G, Matlab R2011b.

### Comparison results of the newly proposed single expression CoPSSM with two other common representations PseAAC and PSSM

To demonstrate effectiveness of the newly proposed single feature expression CoPSSM, we conducted the comparison experimental investigation. Firstly, the often-used 40-D PseAAC and 400-D PSSM were extracted from the given protein sequences respectively. Secondly, the proposed 210-D CoPSSM would be obtained based on PSSM matrix. Thirdly, the KNN classifier with cosine distance was used to predict protein subnuclear location. Last, the Jackknife test, identified as the most objective and rigorous method was utilized to evaluate the classification performance [14]. Concrete prediction accuracy (ACC) for each Class and the overall success rate (OSR) are in Tables 4 and 5.

**Table 4:** Prediction results for protein subnuclear of dataset 1.

Type*	Feature Representation		
	PseAAC (40-D)	PSSM (400-D)	The proposed CoPSSM (210-D)
Class 1	30.2326%	55.8140%	70.9302%
Class 2	36.2832%	41.5929%	53.0973%
Class 3	29.4118%	**0**	23.5294%
Class 4	16.6667%	22.2222%	50%
Class 5	41.6667%	58.3333%	66.6667%
Class 6	32%	30%	26%
Class 7	86.0544%	81.6327%	78.5714%
Class 8	**0**	10%	23.3333%
Class 9	5.4054%	10.8108%	43.2432%
Class 10	**0**	**0**	25%
**OSR**	**52.4663% (K = 9)**	**55.0075% (K = 5)**	**61.5845% (K = 1)**

* Class 1 ~ Class 10 denote Centromere, Chromosome, Nuclear envelope, Nuclear matrix, Nuclear pore complex, Nuclear speckle, Nucleolus, Nucleoplasm, Telomere and Nuclear PML body respectively.

**Table 5:** Prediction results for protein subnuclear of dataset 2.

Type*	Feature Representation		
	PseAAC (40-D)	PSSM (400-D)	The proposed CoPSSM (210-D)
Class 1	48.4848%	69.6970%	59.5960%
Class 2	22.7272%	36.3636%	36.3636%
Class 3	24.5902%	29.5082%	49.1803%
Class 4	13.7931%	31.0345%	44.8276%
Class 5	55.6962%	67.0886%	78.4810%
Class 6	29.8507%	40.2985%	32.8358%
Class 7	81.4332%	71.3355%	79.1531%
Class 8	**0**	16.2162%	18.9189%
Class 9	**0**	7.6923%	15.3846%
**OSR**	**54.0616% (K = 12)**	**57.4230% (K = 3)**	**62.4650% (K = 1)**

* Class 1 ~ Class 9 denote Chromatin, Heterochromatin, Nuclear envelope, Nuclear matrix, Nuclear pore complex, Nuclear speckle, Nucleolus, Nucleoplasm and Nuclear PML body respectively.

From Table 4, it clearly displays that the overall prediction success rate for our newly proposed feature representation CoPSSM outperforms the two most-frequently used expressions PseAAC and PSSM. Not only that, but CoPSSM can resolve the imbalanced data problem. As evident in Table 2, these two benchmark datasets are heavily imbalanced. Generally, classifier tends to be biased towards the majority class, resulting in poor accuracy for those classes having smaller number of samples [33]. For PseAAC, we can find bad prediction for Classes 8 and 10 with accuracy of 0, and this kind of situation still exists in PSSM although its OSR is larger than that of PseAAC. For CoPSSM, even though some prediction accuracies are smaller than those of PseAAC and PSSM for the same Class, there is no bad prediction, which denotes the proposed new feature representation CoPSSM can solve the data imbalance problem to a certain degree. This is because CoPSSM can express protein sequence better than the 40-D PseAAC and the 400-D PSSM.

Table 5 also shows that the proposed CoPSSM performs better than both PseAAC and PSSM. Besides, we can clearly see that bad prediction accuracies for Classes 8 and 9 are all equal to 0 when the feature representation is PseAAC. For CoPSSM, even though several prediction accuracies are smaller than those of PseAAC and PSSM, there is no bad prediction, and the results denote the proposed new feature expression CoPSSM still can resolve the imbalance problem of dataset 2.

### Results of assessment criteria for PseAAC, PSSM and the proposed CoPSSM

Next, to objectively evaluate effectiveness of our proposed new feature representation CoPSSM in protein subnuclear location, we calculated the comparative values of these 4 assessment criteria: SE (Sensitivity), SP (Specificity), ACC (Accuracy) and MCC (Mathew’s Correlation Coefficient) for PseAAC, PSSM and the proposed CoPSSM. What shown in Table 6 is comparison result for dataset 1, and Table 7 is results for dataset 2. Tables 6 and 7 were obtained according to Eqs (8)–(11).

**Table 6:** SE, SP, ACC and MCC for PseAAC, PSSM and the proposed CoPSSM on dataset 1.

Type and Representation		Evaluation Index			
		SE	SP	ACC	MCC
Class 1	PseAAC	0.3023	0.9286	0.8050	0.2859
	PSSM	0.5581	0.8964	0.8307	0.4565
	The proposed CoPSSM	0.7093	0.9117	0.8747	0.5979
Class 2	PseAAC	0.3628	0.7908	0.6950	0.1492
	PSSM	0.4159	0.7716	0.6957	0.1735
	The proposed CoPSSM	0.5310	0.8756	0.8	0.4106
Class 3	PseAAC	0.2941	0.9774	0.9461	0.3088
	PSSM	**0**	0.9866	0.9436	-0.0243
	The proposed CoPSSM	0.2353	0.9831	0.9537	0.2696
Class 4	PseAAC	0.1667	0.9943	0.9538	0.2999
	PSSM	0.2222	0.9918	0.9558	0.3382
	The proposed CoPSSM	0.5	0.9805	0.9604	0.4939
Class 5	PseAAC	0.4167	0.9914	0.9723	0.4969
	PSSM	0.5833	0.9863	0.9735	0.5697
	The proposed CoPSSM	0.6667	0.9902	0.9810	0.6569
Class 6	PseAAC	0.32	0.9571	0.8775	0.3428
	PSSM	0.3	0.9671	0.8867	0.3526
	The proposed CoPSSM	0.26	0.9236	0.8548	0.1905
Class 7	PseAAC	0.8605	0.3590	0.6190	0.2550
	PSSM	0.8163	0.4830	0.6583	0.3190
	The proposed CoPSSM	0.7857	0.7269	0.7587	0.5135
Class 8	PseAAC	**0**	1	0.9213	**-**
	PSSM	0.1	0.9892	0.9223	0.1791
	The proposed CoPSSM	0.2333	0.9485	0.9015	0.1847
Class 9	PseAAC	0.0541	0.9831	0.8954	0.0768
	PSSM	0.1081	0.9945	0.9132	0.2447
	The proposed CoPSSM	0.4324	0.9451	0.9035	0.3686
Class 10	PseAAC	**0**	0.9943	0.9616	-0.0137
	PSSM	**0**	0.9973	0.9659	-0.0093
	The proposed CoPSSM	0.25	0.9808	0.9604	0.2408

**Table 7:** SE, SP, ACC and MCC for PseAAC, PSSM and the proposed CoPSSM on dataset 2.

Type and Representation		Evaluation Index			
		SE	SP	ACC	MCC
Class 1	PseAAC	0.4848	0.7897	0.7324	0.2439
	PSSM	0.6970	0.7445	0.7361	0.3579
	The proposed CoPSSM	0.5960	0.9021	0.8447	0.4943
Class 2	PseAAC	0.2273	0.9896	0.9484	0.3335
	PSSM	0.3636	0.9617	0.9318	0.3123
	The proposed CoPSSM	0.3636	0.9648	0.9370	0.3151
Class 3	PseAAC	0.2459	0.9789	0.8773	0.3490
	PSSM	0.2951	0.9561	0.8705	0.3174
	The proposed CoPSSM	0.4918	0.9455	0.8902	0.4611
Class 4	PseAAC	0.1379	0.9922	0.9324	0.2576
	PSSM	0.3103	0.9733	0.9297	0.3379
	The proposed CoPSSM	0.4483	0.9752	0.9429	0.4629
Class 5	PseAAC	0.5570	0.8930	0.8355	0.4372
	PSSM	0.6709	0.952	0.9031	0.6501
	The proposed CoPSSM	0.7848	0.9576	0.9292	0.7424
Class 6	PseAAC	0.2985	0.9632	0.8635	0.3523
	PSSM	0.4030	0.9341	0.8595	0.3697
	The proposed CoPSSM	0.3284	0.9319	0.8544	0.2882
Class 7	PseAAC	0.8143	0.4533	0.6359	0.2874
	PSSM	0.7134	0.6945	0.7045	0.4076
	The proposed CoPSSM	0.7915	0.6767	0.7348	0.4716
Class 8	PseAAC	**0**	0.9897	0.9040	-0.03
	PSSM	0.1622	0.9735	0.9071	0.1955
	The proposed CoPSSM	0.1892	0.9543	0.8974	0.1634
Class 9	PseAAC	**0**	1	0.9674	**-**
	PSSM	0.0769	0.9951	0.9670	0.1482
	The proposed CoPSSM	0.1538	0.9801	0.9571	0.1453

In Table 6, we can learn that there exist such outliers of 0 and “-” for the expressions PseAAC and PSSM, where “-” means the missing phenomenon in computation. According to Eqs (8) and (11), it can be inferred that none of these types of proteins were correctly located and no other type of proteins was misclassified to them, which caused the exceptional values 0 and “-”, respectively. Indeed, the results in Table 4 confirm this conclusion. In Table 6, we can see most values of CoPSSM are larger than those of PseAAC and PSSM for the same Class, which signifys that CoPSSM outperforms them.

For the outliers 0 and “-” in Table 7, the explanation is the same as that of Table 6, and the results in Table 5 can confirm it. Similarly, we can conclude that the proposed feature representation CoPSSM performs better than both PseAAC and PSSM in protein subnuclear localization.

### The overall success rate (OSR) of PseAAC, PSSM and the proposed CoPSSM for different K values of KNN classifier

**Fig 3:**
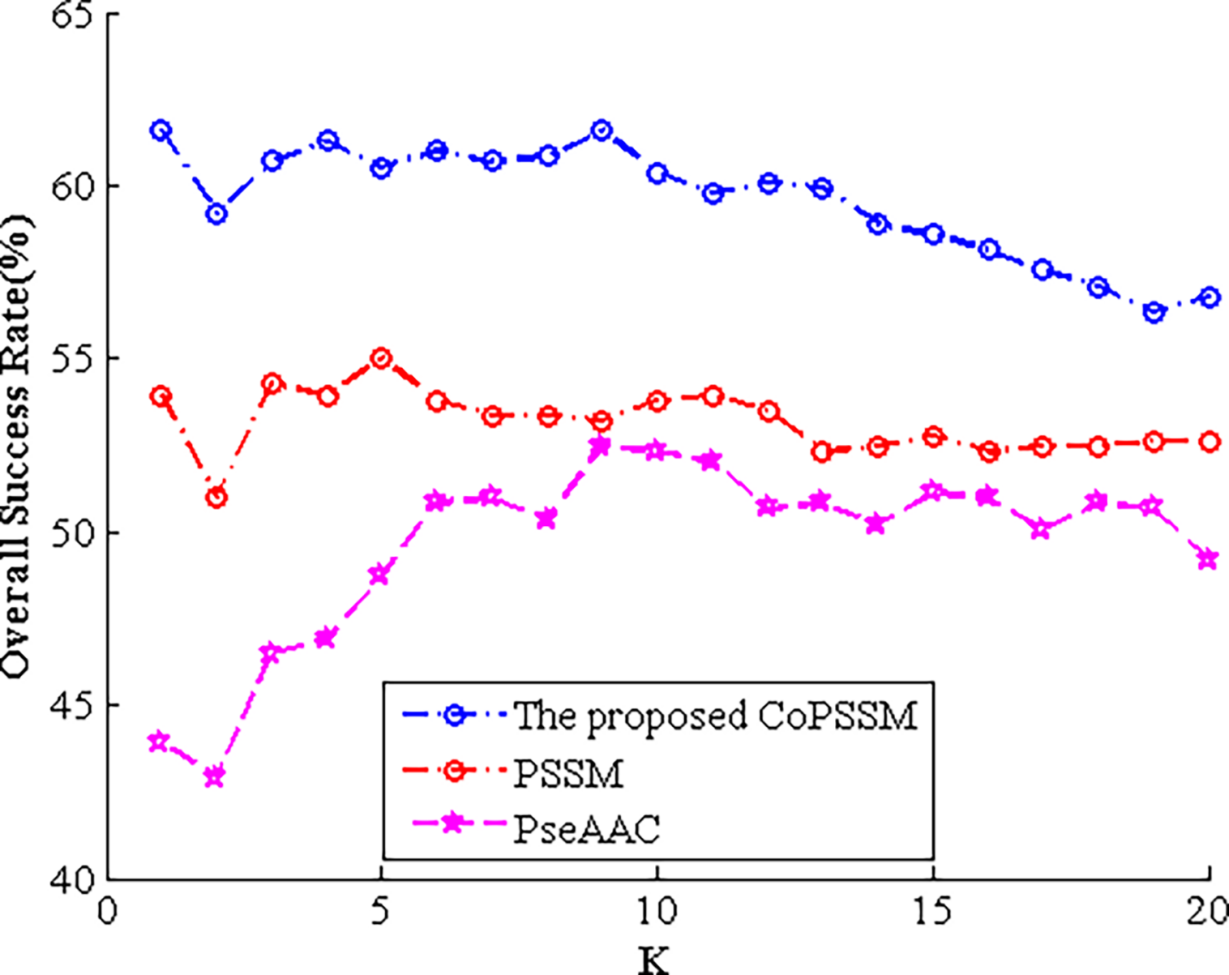
The OSR of PseAAC, PSSM and the proposed CoPSSM for different K values on dataset 1.

**Fig 4:**
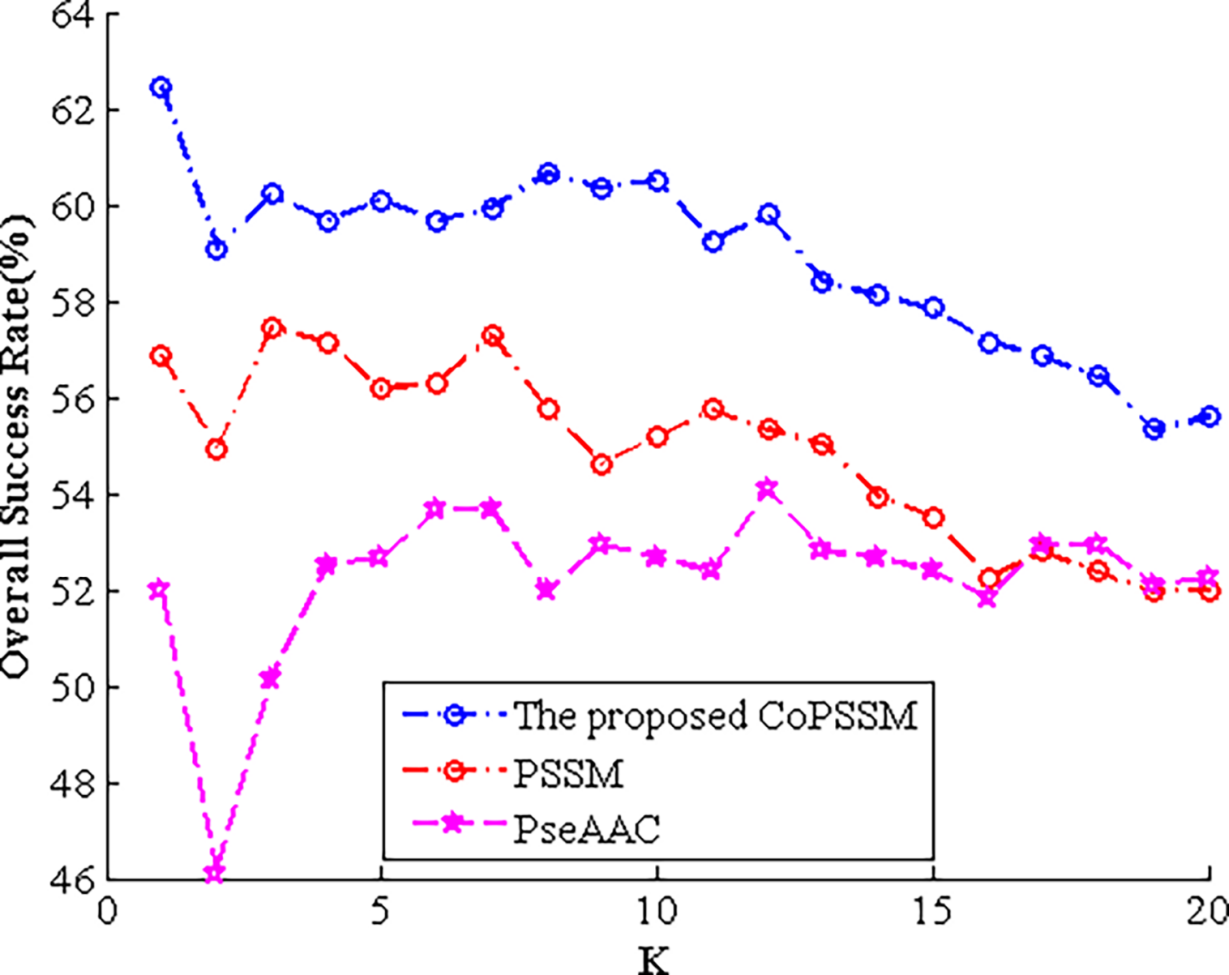
The OSR of PseAAC, PSSM and the proposed CoPSSM for different K values on dataset 2.

Since various K values of the k-nearest-neighbour have an effect on the prediction performance, here what we displayed in Tables 4 and 5 are the highest OSR among different K values. Therefore, we’ll provide following Figs 3 and 4 to show corresponding OSR of PseAAC, PSSM and the proposed CoPSSM when K ranges from 1 to 20.

Figs 3 and 4 clearly display that the newly proposed feature representation CoPSSM performs the best, the often used 400-D PSSM is in second place and PseAAC is the last. From Fig 3, we can find that when K is equal to 9, 5 and 1, expressions PseAAC, PSSM and CoPSSM obtain their biggest prediction accuracy, respectively. For dataset 2, corresponding K values are 12, 3 and 1 separately.

### Prediction results of the proposed method: CoPSSM with intelligent KLDA based on DGGA

Based on above analysis, we have proved that the newly proposed feature representation CoPSSM outperforms the two most frequently used representations, PseAAC and PSSM. Since an effective dimension reducing method played significant role in the prediction of protein subnuclear location [4, 12, 13], here kernel linear discriminant analysis (KLDA) was applied to conduct dimensionality reduction on the proposed and best-performing feature expression CoPSSM. Kernel function of KLDA is the key point and it has a great influence on the final results. However, there is short of rational method to select the optimal kernel parameter of kernel function at present. Inspired by it, we proposed a new algorithm based on the genetic algorithm (GA) to intelligently search the optimal kernel parameter. In the meantime, a new discriminant criterion was put forward to serve as fitness of the proposed optimization algorithm, dichotomous greedy genetic algorithm (DGGA). Hence, we named this method as CoPSSM with intelligent KLDA based on DGGA. Last, the dimension-reduced CoPSSM would be taken as input of KNN classifier to predict protein subnuclear location.

Here, the kernel linear discriminant analysis (KLDA) algorithm was implemented in MATLAB (R2011b version), using the famous Matlab Toolbox (developed by Laurens van der Maaten, Delft University of Technology).

Different dimensionalities of the reduced data would influence the prediction results of protein subnuclear location, and since the Jackknife test method was very time-consuming, we took the defaulted value of KLDA as the reduced dimension with a view to the operating convenience and conciseness. Namely, the number of protein type was set to the reduced dimension. Hence the reduced dimensionality for datasets 1 and 2 were 10 and 9 respectively. Prediction results are as Table 8.

**Table 8:** Prediction results of the proposed method.

	Dataset 1 (reduced 10-D CoPSSM)	Dataset 2 (reduced 9-D CoPSSM)
Class 1	72.0930%	84.8485%
Class 2	80.5310%	90.9091%
Class 3	100%	86.8852%
Class 4	94.4444%	89.6552%
Class 5	83.3333%	88.6076%
Class 6	98%	89.5522%
Class 7	92.1769%	93.1596%
Class 8	93.3333%	91.8919%
Class 9	75.6757%	92.3077%
Class 10	100%	
**OSR**	**87.4440% (K = 13)**	**90.3361% (K = 10)**

From Table 8, we can clearly see that the overall success rates (OSR) for datasets 1 and 2 are 87.444% and 90.3361% respectively. Besides, prediction accuracy for each class is no less than 70% and even can reach up to 100% for Classes 3 and 10 on dataset 1. For dataset 2, there’s narrower fluctuation margin for each class. This is probably because the inherent attributes of different datasets lead to such a result. To show effectiveness of the proposed method, in the next section, it will be extended to predict protein subcellular localization.

### Experimental development: Predicting protein subcellular location via the proposed method, CoPSSM with intelligent KLDA based on DGGA

To show the generalization of the proposed method, CoPSSM with intelligent KLDA based on DGGA, two protein subcellular benchmark datasets (datasets 3 and 4, shown in Table 3) were chosen to conduct the numerical experiment. Furthermore, dataset 4 was used as the real validation set of dataset 3 instead of the Jackknife test; namely, as the training set, dataset 3 was tested by the Jackknife method while dataset 4 as the testing set was tested by the Independent method. Still, we firstly verified the newly proposed feature representation CoPSSM outperforms the commonly used 40-D PseAAC and 400-D PSSSM. In the second place, the proposed method CoPSSM with intelligent KLDA based on DGGA was employed to predict protein subcellular location to demonstrate its effectiveness. The detailed experimental results are as Tables 9 and 10.

**Table 9:** Prediction results for protein subcellular location.

Type *	Jackknife test for dataset 3			Independent test for dataset 4		
	PseAAC (40-D)	PSSM (400-D)	CoPSSM (210-D)	PseAAC (40-D)	PSSM (400-D)	CoPSSM (210-D)
Class 1	90.7895%	86.8421%	94.0790%	90.4762	89.0476	88.5714
Class 2	35.5263%	53.9474%	55.2632%	50	65	60
Class 3	**0**	8.3333%	50%	**0**	**0**	75
Class 4	**0**	83.3333%	83.3333%	100	100	100
Class 5	84.9462%	86.0215%	86.0215%	77.9710	79.1304	78.5507
Class 6	**0**	**0**	**0**	**0**	**0**	**0**
Class 7	60.1942%	54.3689%	61.1651%	46.1539	76.9231	84.6154
Class 8	48.2143%	65.1786%	53.5714%	61.2245	65.3061	57.1429
**OSR**	**67.2282% (K = 5)**	**71.6692% (K = 8)**	**73.3538% (K = 6)**	**78.6936%** **(K = 6)**	**80.2488%** **(K = 12)**	**79.6268%** **(K = 5)**

* Class 1 ~ Class 8 denote Cytoplasm, Extracell, Fimbrium, Flagellum, Inner membrane, Nucleoid, Outer membrane, Periplasm respectively.

**Table 10:** Prediction results of the proposed method for protein subcellular location.

Type	Jackknife test for dataset 3	Independent test for dataset 4
	Reduced 8-D CoPSSM	Reduced 8-D CoPSSM
Class 1	136/152 = 89.4737%	186/210 = 88.5714%
Class 2	67/76 = 88.1579%	18/20 = 90%
Class 3	10/12 = 83.3333%	4/4 = 100%
Class 4	6/6 = 100%	1/1 = 100%
Class 5	181/186 = 97.3118%	339/345 = 98.2609%
Class 6	6/6 = 100%	1/1 = 100%
Class 7	97/103 = 94.1748%	12/13 = 92.3077%
Class 8	100/112 = 89.2857%	48/49 = 97.9592%
**OSR**	603/653 = **92.3430% (K = 13)**	609/643 = **94.7123% (K = 5)**

From Table 9, we can get some useful information. For the Jackknife test dataset 3, it clearly indicates that the proposed new feature representation CoPSSM performs better than the commonly used 40-D PseAAC and 400-D PSSM in discriminant ability. In addition, it shows CoPSSM can solve the data imbalance problem for Classes 3 and 4 to a certain degree as well. For the Independent test dataset 4, the performance of CoPSSM is superior to the 40-D PseAAC while is inferior to the 400-D PSSM; however, we still recommend the proposed CoPSSM in view of its lower dimensionality and better discriminant ability in dealing with the data imbalance problem than the 400-D PSSM. Despite all this, we can find that the proposed CoPSSM still can’t predict proteins belonging to Class 6, which requires us to improve this feature expression in the future work.

Next, the proposed method CoPSSM with intelligent KLDA based on DGGA will be utilized to predict protein subcellular location. Numerical experiment results are shown in Table 10.

From Table 10, we can clearly see that the overall success rates (OSR) for the training dataset 3 and the testing dataset 4 are 92.3430% and 94.7123% respectively, which proves the generalization of the proposed method in protein subcellular location. Last, to show effectiveness of the proposed method CoPSSM with intelligent KLDA based on DGGA, it’s necessary to compare it with state-of-the-art predictors on the same benchmark datasets. In the next section, detailed comparison results can justify efficiency of the proposed method in protein subnuclear and subcellular localization.

### Compare with existing prediction results

Table 11 clearly shows comparison results of the overall success rate among the proposed method and different state-of-the-art algorithms on the four standard datasets.

**Table 11:** Comparison of overall success rate on the four benchmark datasets.

		Algorithm	Representation and method	OSR (%)
Dataset 1		SubNucPred [33]	SSLD and AAC based on SVM by Jackknife test	81.46
		Effective Fusion Representations [4]	DipPSSM with LDA based on KNN by 10-fold cross-validation	≈97
			PseAAPSSM with LDA based on KNN by 10-fold cross-validation	≈84
		**The proposed method: CoPSSM with intelligent KLDA based on DGGA**	CoPSSM with KLDA based on KNN and Jackknife test	**87.44**
Dataset 2		Nuc-PLoc [7]	Fusion of PsePSSM and PseAAC based on Ensemble classifier by Jackknife test	67.4
		Effective Fusion Representations [4]	DipPSSM with LDA based on KNN by 10-fold cross-validation	95.94
			PseAAPSSM with LDA based on KNN by 10-fold cross-validation	88.1
		**The proposed method: CoPSSM with intelligent KLDA based on DGGA**	CoPSSM with KLDA based on KNN and Jackknife test	**90.34**
Dataset 3	Gneg-PLoc [34]		Fusion of GO approach and PseAAC based on Ensemble classifier by Jackknife test	87.3
	Nonlinear dimensionality reduction method [12]		Fusion of PSSM and PseAAC with KLDA based on KNN by Jackknife test	98.77
	**The proposed method: CoPSSM with intelligent KLDA based on DGGA**		CoPSSM with KLDA based on KNN and Jackknife test	**92.34**
Dataset 4	Gneg-PLoc [34]		Fusion of GO approach and PseAAC based on Ensemble classifier by Independent test	89.3
	**The proposed method: CoPSSM with intelligent KLDA based on DGGA**		CoPSSM with KLDA based on KNN and Independent test	**94.71**

The most significant aspect that Table 11 reveals is that our proposed optimization algorithm can achieve good effect only with the single feature vector CoPSSM in the prediction of protein subnuclear and subcellular localization; whereas the other researches employed complex multiple representations, for instance, by fusing two kinds of different single representations. For dataset 1, our proposed method outperforms the SubNucPred [33] and the second method in [4]. However, it is inferior to the first method in [4]. For dataset 2, the proposed method prevails over the Nuc-PLoc [7] and the second method in [4] while is still inferior to its first method. Note that the Jackknife test method used by us is more rigorous than the 10-fold cross-validation test employed in [4]. Hence, it reveals that our method is effective and meaningful, and at the same time, it also reveals that prediction efficiency of single feature representation is less than that of information-rich fusion representation. For the extended experiment (predicting protein subcellular localization), results of dataset 3 and 4 still show the proposed method is effective. Besides, it proves again that the fusion representation contains more protein sequence information than the single feature expression, which makes the former get higher prediction result than the latter.

### Conclusions

Until now, numerous studies have discussed protein subnuclear location [46–48]. Simultaneously, prediction accuracy is getting higher and higher with newly developed methods and techniques [31, 49–52]. However, design complexity and time-consuming that come with it are still thorny problems that need to be addressed. Taking into these issues consideration, this paper tactfully put forward a new feature representation and idea to identify the protein subnuclear location. First, a more informative feature expression CoPSSM was created based on PSSM. Second, to search the bandwidth parameter of gauss kernel intelligently, we proposed an optimization algorithm DGGA based on GA. Third, to evaluate results of dimension reduction, we proposed a new discriminant criterion as the fitness of DGGA. Compared with other people’s research findings, our method can get high performance partly.

To verify generality and validity of the proposed method, two protein subnuclear standard datasets and Jackknife test, were considered to conduct the numerical experiments. Then, SE, SP, ACC and MCC were taken as the evaluation indexes. The experimental results were encouraging. Furthermore, the proposed method with Independent test was utilized to predict protein subcellular location and the obtained results still demonstrated its effectiveness. Whereas the dimensionality reduction algorithms KLDA undoubtedly adds the computational complexity to a certain degree, therefore, whether we can directly make good predictions without employing any dimensionality reduction algorithm deserves us to have thorough analysis and study in the future work. In addition, it remains an interesting challenge to obtain better representations for protein subnuclear localization and to study other machine learning classification algorithms [4].

## Supporting information

S1 File Detailed description for KLDA algorithm. (DOCX)
