# Supplementary material for: Protein subnuclear localization based on a new effective representation and intelligent kernel linear discriminant analysis by dichotomous greedy genetic algorithm
Source: PLoS One. 2018 Apr 12;13(4):e0195636. doi: 10.1371/journal.pone.0195636 (PMC5896989; doi:10.1371/journal.pone.0195636)
Supplement: S1 File — (DOCX) [file pone.0195636.s001.docx]

**Detailed description for KLDA algorithm**

Assume that the sample dataset $X$ in input space contains $N$ proteins and $X$ is a union of $k$ kinds of different classes, i.e., $X=C_{1}\cup C_{2}\ldots\cup C_{k}=\left\{ x_{1},x_{2},\ldots,x_{N} \right\},$where$C_{i}=\left\{ x_{i1},x_{i2},\ldots,x_{{iN}_{i}} \right\}$ $(i=1,2,\ldots,k.)$, and $N_{i}$ is the number of class$C_{i}$, thus$N=\sum_{i=1}^{k} N_{i}$. Then, the input vectors: $x_{1},x_{2},\ldots,x_{N}$ are all mapped to $\emptyset(x_{1}),\emptyset(x_{2}),\ldots,\emptyset(x_{N})\in F$ via the nonlinear mapping function$\emptyset$. Next, we can obtain these following formulas in feature space $F$ according to the theory of LDA.

$m_{i}^{\emptyset}=\frac{1}{N_{i}}\sum_{x\in C_{i}} \emptyset\left( x \right)$ $(i=1,2,\ldots,k.)$ (1)

$m^{\emptyset}=\frac{1}{N}\sum_{i=1}^{k} \sum_{x\in C_{i}} \emptyset(x)$ (2)

Here, $m_{i}^{\emptyset}$ is mean of the mapping samples for class $C_{i}$ and $m^{\emptyset}$ is mean of all the data in the new feature space. With that, we can obtain the within-class scatter matrix for each class, the within-class covariance matrix and the between-class covariance matrix for the whole samples respectively, which are named$S_{i}^{\emptyset} (i=1,2,\ldots k)$, $S_{w}^{\emptyset}$ and $S_{b}^{\emptyset}$ respectively.

$S_{i}^{\emptyset}=\sum_{x\in C_{i}} {[\emptyset\left( x \right)-m_{i}^{\emptyset}][\emptyset\left( x \right)-m_{i}^{\emptyset}]}^{T} \left( i=1,2,\ldots k \right)$ (3)

$S_{w}^{\emptyset}=\sum_{i=1}^{k} S_{i}^{\emptyset}=\sum_{i=1}^{k} \sum_{x\in C_{i}} {[\emptyset\left( x \right)-m_{i}^{\emptyset}][\emptyset\left( x \right)-m_{i}^{\emptyset}]}^{T}$ (4)

$S_{b}^{\emptyset}=\sum_{i=1}^{k} N_{i}{\left[ m_{i}^{\emptyset}-m^{\emptyset} \right]\left[ m_{i}^{\emptyset}-m^{\emptyset} \right]}^{T}$ (5)

Suppose that $w$ is the projection direction in feature space$F$. Then, the dimension reduction criterion function, which minimizes difference of within subclasses and maximizes difference between each class, is as below:

$\max J_{F}\left( w \right)=\frac{w^{T}S_{b}^{\emptyset}w}{w^{T}S_{w}^{\emptyset}w}$ (6)

Here, what we need to do is to work out the most optimal direction of projection$w^{*}$, by the methods of generalized eigen-decomposition. However, it’s impossible for us to solve them, since the mapping function $\emptyset$ is unknown and all of the formulas above are in the feature space$F$, which has high dimensionality even infinite. Therefore, kernel trick is introduced to solve this problem.

$K\left( x,y \right)=<\emptyset\left( x \right),\emptyset\left( y \right)>$ (7)

Note that $w$ could be the linear combination of$\emptyset\left( x_{1} \right),\emptyset\left( x_{2} \right),\ldots,\emptyset(x_{N})$, namely

$w=\sum_{i=1}^{N} a_{i}\emptyset\left( x_{i} \right)$ (8)

Then, we can obtain equations (9) and (10) according to equations (1), (2) and (7).

$w^{T}m_{i}^{\emptyset}=\frac{1}{N_{i}}\sum_{j=1}^{N} \sum_{n=1}^{N_{i}} a_{j}K\left( x_{j},x_{n} \right)=a^{T}M_{i}$ (9)

$w^{T}m^{\emptyset}=\frac{1}{N}\sum_{j=1}^{N} \sum_{n=1}^{N} a_{j}K\left( x_{j},x_{n} \right)=a^{T}M$ (10)

where,$a={(a_{1},a_{2},\ldots,a_{N})}^{T}, M_{i}$ and $M$ are as formulas (11) and (12):

${(M_{i})}_{j}=\frac{1}{N_{i}}\sum_{n=1}^{N_{i}} K\left( x_{j},x_{n} \right) \left( i=1,2,\ldots,k; j=1,2,\ldots,N \right)$ (11)

${(M)}_{j}=\frac{1}{N}\sum_{n=1}^{N} K\left( x_{j},x_{n} \right) (j=1,2,\ldots,N)$ (12)

Therefore, equations (13) and (14) can be formulized as follows on the basis of above equations (4), (5), (8), (9) and (10).

$w^{T}S_{b}^{\emptyset}w=a^{T}\tilde{M}a$ (13)

$w^{T}S_{w}^{\emptyset}w \triangleq a^{T}\tilde{L}a$ (14)

Here in the above formulas$, \tilde{M}=\sum_{i=1}^{k} N_{i}\left( M_{i}-M \right)\left( M_{i}-M \right)^{T}, \tilde{L}=\sum_{i=1}^{k} K_{i}(I-1_{N_{i}}){K_{i}}^{T}$. And $K_{i}$ is the kernel matrix for class$i$, with${(K_{i})}_{N\times N_{i}}=K\left( x, x_{i} \right) (x=\left( x_{1},x_{2},\ldots,x_{N} \right); x_{i}\in C_{i})$.

Thus, the finally dimension reduction criterion function can be rewritten as (15):

$\max J_{(a)}=\frac{a^{T}\tilde{M}a}{a^{T}\tilde{L}a}$ (15)

According to [40, 41], for multi-class pattern classification, such as $k$ classification problems of this paper, the orthonormal columns of $a^{*}$ must satisfy equation (16), which is a generalized eigenvalue problem.

$\tilde{M}a_{i}=\gamma_{i}\tilde{L}a_{i} \left( i=1,2,\ldots,k \right)$ (16)

Hence, the eigenvectors of $\tilde{L}^{-1}\tilde{M}$ corresponding to the largest $k$ eigenvalues are the columns of the optimal projection matrix $a^{*}$on the condition that $\tilde{L}$ is nonsingular. And, we can obtain the finally rank-reduction projective matrix $Y$ through formula (17):

$Y={{(a}^{*})}^{T}X$ (17)
